# *Borrelia burgdorferi* aggrecanase activity: more evidence for persistent infection in Lyme disease

**DOI:** 10.3389/fcimb.2013.00040

**Published:** 2013-08-14

**Authors:** Raphael B. Stricker, Lorraine Johnson

**Affiliations:** International Lyme and Associated Diseases SocietyBethesda, MD, USA

**Keywords:** Lyme disease, *Borrelia burgdorferi*, aggrecanase, arthritis, BbHtrA

## Abstract

Lyme disease is the most common tickborne illness in the world today. A recent study describes for the first time an enzyme produced by the spirochetal agent of Lyme disease, *Borrelia burgdorferi*, that cleaves aggrecan, a proteoglycan found in joints and connective tissue. Discovery of the spirochetal aggrecanase raises many questions about the pathogenesis of Lyme arthritis and lends support to the concept of persistent *B. burgdorferi* infection in patients with chronic Lyme disease symptoms.

Lyme disease is the most common tickborne illness in the world today (Stricker and Johnson, 2011). As of July 2013, the Medline database lists more than 25,000 published peer-reviewed articles about tickborne diseases. Despite this plethora of scientific information, however, the pathogenetic mechanisms of rheumatological, neurological and cardiac complications induced by the Lyme spirochete, *Borrelia burgdorferi*, have not been fully elucidated (Schmit et al., 2011; Lin et al., 2012). In particular, the mode of tissue invasion by the Lyme spirochete remains obscure.

In an elegant study of a potential mechanism of Lyme arthritis, Russell and Johnson describe for the first time an enzyme produced by *B. burgdorferi* that cleaves aggrecan, a proteoglycan found in joints and connective tissue (Russell and Johnson, 2013). The spirochetal aggrecanase, called BbHtrA, was identified in the three major species of *B. burgdorferi*, was expressed on the spirochete surface, and was shown to cleave aggrecan at a site that destroys the function of the proteoglycan. Furthermore, and perhaps of greatest importance, the proteoglycan degradation fragments produced by BbHtrA cleavage have been detected in the synovial fluid of patients with Lyme arthritis. These observations suggest that the spirochetal enzyme plays a role in the rheumatological pathology of Lyme disease (Russell and Johnson, 2013).

Although the discovery of BbHtrA suggests a novel spirochetal mechanism of Lyme arthritis, the involvement of aggrecanase in this process is not novel. A previous study by Behera et al. showed that *B. burgdorferi* may induce expression of ADAMTS-4, a host tissue aggrecanase that can cleave the proteoglycan in the joints of mice and humans (Behera et al., 2006). Thus, the Lyme spirochete may induce joint damage using both host tissue (ADAMTS-4) and spirochetal (BbHtrA) aggrecanases. Although Russell and Johnson touch briefly on this point in their report, the implications of the redundant enzyme activity merit further discussion.

Aggrecanase activity has been associated with various forms of arthritis and more recently with progression of degenerative disc disease and aortic aneurysm dissection (Huang and Wu, 2008; Mimata et al., 2012; Tian et al., 2013; Zhang et al., 2013). The principal tissue aggrecanases ADAMTS-4 and ADAMTS-5 appear to be regulated by inflammatory cytokines such as IL-6, TNF-α and IL-1β, and these enzymes appear to work in tandem to induce joint injury (Mimata et al., 2012; Tian et al., 2013). Based on their ability to damage proteoglycans, the tissue aggrecanases have been identified as potential therapeutic targets in the prevention and treatment of arthritis and connective tissue diseases (Huang and Wu, 2008; Mimata et al., 2012; Ren et al., 2013; Tian et al., 2013; Zhang et al., 2013). As pointed out by Russell and Johnson, secreted aggrecanases from several gram-negative bacteria also appear to be involved in tissue invasion and propagation of infection under varying stress conditions in the host (Wu et al., 2011; Hoy et al., 2013).

The discovery of a spirochetal aggrecanase raises significant questions about the mechanisms of joint and connective tissue injury as well as tissue invasion by *B. burgdorferi*. How does this pathogen-associated enzyme work in conjunction with the tissue aggrecanases, and which one is dominant? How is the spirochetal enzyme regulated, and specifically do inflammatory cytokines influence expression of the spirochetal aggrecanase in the same manner as the tissue enzymes? Are the aggrecan cleavage sites the same for the tissue and spirochetal enzymes? Do tickborne coinfections influence expression of the spirochetal aggrecanase, as suggested by previous studies of tissue enzyme activity (Grab et al., 2007)? And as with other bacteria that secrete a similar aggrecanase, do these enzymes play a more general role in connective tissue invasion by *B. burgdorferi*, including induction of disease in other organ systems? The answers to these and related questions will help to elucidate both the mechanisms of acute Lyme arthritis and the evolution of persistent infection with the Lyme spirochete (Berndtson, 2013; Stricker and Johnson, 2013).

Controversy persists over the existence of chronic Lyme disease due to persistent infection with *B. burgdorferi* in patients who are untreated or undertreated for the spirochetal illness (Berndtson, 2013; Stricker and Johnson, 2013). While some researchers continue to insist that there is no “credible scientific evidence” for chronic Lyme disease, a growing body of clinical and research evidence supports persistent symptomatic infection with the Lyme spirochete (Miklossy, 2012; Berndtson, 2013; Ljøstad and Mygland, 2013; Stricker and Johnson, 2013). The discovery of a spirochetal aggrecanase that damages tissues and enhances tissue invasion by *B. burgdorferi* adds support to the concept of persistent symptomatic infection in Lyme disease. The extent to which this spirochetal enzyme helps *B. burgdorferi* to penetrate tissue sites, evade the immune response and survive antibiotic therapy remains to be determined.

The study by Russell and Johnson is yet another reminder of the complexity of *B. burgdorferi* pathogenesis (Salman-Dilgimen et al., 2011; Chaconas, 2012; Chaconas and Norris, 2013). The fact that the Lyme spirochete can apparently utilize its own enzyme as well as hijacking a host enzyme to facilitate tissue invasion underscores the regulatory and molecular complexity of the spirochete, which contains one of the “most unusual genomes on the planet” (Chaconas, 2012). From a microbiological perspective, the study of *B. burgdorferi* gene function is yielding fascinating clues about the interaction of this organism with its host and the coordinated interplay within its own genetic elements (Salman-Dilgimen et al., 2011; Schmit et al., 2011; Chaconas, 2012; Chaconas and Norris, 2013). Clearly we still have lots to learn about the science of Lyme disease (Pearson and Huyshe-Shires, 2013).

## Conflict of interest statement

Raphael B. Stricker serves without compensation on the medical advisory panel of QMedRx Inc. He has no financial ties to the company. Lorraine Johnson has no conflicts to declare. The work is original, and both authors had a role in writing the manuscript.
